# AVATS: Awake Video Assisted Thoracic Surgery –extended series report

**DOI:** 10.1186/s13019-014-0149-x

**Published:** 2014-08-28

**Authors:** Ara S Klijian, Michael Gibbs, Nicole T Andonian

**Affiliations:** Department of Cardiothoracic Surgery at Sharp & Scripps Healthcare, 3131 Berger Ave, Suite 250, San Diego, 92123 CA USA; Department of Anesthesia at Sharp Coronado Hospital, San Diego, USA

**Keywords:** Awake VATS, Local, Sedation thoracoscopy

## Abstract

**Background:**

Traditionally, video-assisted thoracic surgery (VATS) is performed under general anesthesia with selective ventilation and endotracheal intubation. Although some sparse data exists on VATS under local anesthesia, most series reserve this technique for pleural-based surgery. Performing VATS under local anesthesia may extend the benefits of this procedure to those unable to tolerate general anesthesia and improve outcomes.

**Method:**

We have extended this technique to include more complex procedures, with results that surpass traditional open thoracotomies. We analyzed 293 patients who underwent awake video-assisted thoracic surgery (AVATS) from June 2010 to January 2014.

**Results:**

Procedures such as pleural biopsies, wedge resections, decortications, and even lobectomies were able to be safely performed using AVATS technique with comparable or better results than VATS under general anesthesia.

**Conclusion:**

AVATS is a feasible technique with equal or improved outcomes without compromise in safety. Further study may help delineate the role of this technique in the care of the thoracic surgical patient.

## Background

Using local anesthesia with sedation in VATS surgeries minimizes many risks and lets patients breathe spontaneously [1]. This allows for operation on patients with low lung function who cannot tolerate endotracheal intubation or are deemed high risk for general anesthesia surgical procedures [2]. It is particularly useful in patients whose general condition has deteriorated or is poor [3].

Pompeo et al. [4] conducted a study comparing VATS bullectomy and pleural abrasion procedure using local and general anesthesia in patients with spontaneous pneumothorax. The local awake procedure resulted in lower costs and shorter post-operative hospital stays with comparable clinical outcomes to general anesthesia patients. Lesser [5] showed significantly shorter anesthesia time, global operating room time, and hospital stays in local anesthesia patients. Chest incision is limited for awake surgeries but this less invasive approach results in better cosmetic results and minimal trauma.

Using local anesthesia with sedation is best to monitor patient’s physical state and maintain communication [4],[5]. Other advantages include the patient’s ability to cough throughout the procedure, allowing for increased ventilation, less respiratory complications and shorter recovery time [4]. Vanni et al. [6] also found that patients who underwent local anesthesia VATS versus general anesthesia procedures had higher lymphocyte and natural-killer cell count one day after operation. Local anesthesia is not as traumatic for the immune system, allowing for more rapid recovery.

The drawbacks to awake VATS surgery are minimal. Total lung collapse isn’t possible, since patients are breathing spontaneously. A calm surgical environment is essential to minimize stress and anxiety of the patient and increase their comfort level.

## Methods

We analyzed 293 patients who underwent awake thoracic surgery from June 2010 to January 2014. This single surgeon experience includes wedge resections, lobectomies, decortications, pleural biopsies, pleurodesis, bullectomies and pericardial windows. The procedures followed the guidelines of the internal hospital ethics committees. *During the same time period, 183 classic VATS procedures and 71 open thoracotomies were also performed.*

## Results

This series reports a single-surgeon, multi-center review of the AVATS technique. Institutional ethical approval and individual informed consent was obtained from all patients prior to surgery. In total, 293 thoracic surgeries were performed under local anesthesia with sedation between June 2010 and January 2014. Patients ranged from eighteen to ninety years of age, with a mean age of 66. No deaths occurred in the series. Of the 293 cases, ninety-two were wedge resections, with an average length of hospital stay (ALS) of 1.5 days (see Table 1). *Most of these wedge resections were performed for diagnosis of small nodules not amenable for fine needle biopsy, diagnosis of processes such as pulmonary fibrosis, benign inflammatory/infectious processes, i.e. fungal masses, and resection of solitary metastatic melanoma or sarcoma.*

**Table 1:** **Procedures performed and average length of stay**

Cases	Number	ALS
Total	293	
Wedge Resections	92	1.5
Lobectomies	32	1.6
Decortications	68 (8 concurrent wedges)	2.3
Pleural Biopsies	33	1
Pleurodesis	56	1.4
Pericardial Windows	2	1.2
Bullectomies	10	1.4

*Of the 68 decortications performed, all were A2 effusions (>50% involvement of the hemithorax. Eight of these were for early phase empyema, 18 for intermediate phase, and 42 for organized/late phase empyemas. Twenty-eight of these decortications were Category 3 (>50% hemithorax involvement with associated pleural peel/thickening and positive cultures or gram stain, pH, 7.2 and/or pleural glucose >60 mg/dl.) Forty of the decortications were Category 4 (associated with frank pus.)* ALS for the 68 decortications (eight with concurrent wedge resection) was 2.5 days, for the 33 pleural biopsies was 1 day.

There were 56 mechanical and talc pleurodesis for recurrent effusions, 40 of them malignant, having an ALS of 1.4 days. Two pericardial windows were performed, with an ALS of 1.5 days. Thirty-two patients had lobectomies for malignancies (6 left upper lobes, 7 left lower lobes, 5 right upper lobes, 1 right middle lobe, 10 right lower lobes and 1 left lower lobe with lingulectomy), with an ALS of 1.6 days.

Of the 293 patients, only 42 required a one-day ICU stay. The remaining 251 patients went to the post-anesthesia care unit then to a telemetry floor, not requiring the ICU. Comorbidities included 111 patients with diabetes mellitus, 114 patients with chronic obstructive pulmonary disease, 30 with atrial fibrillation, and 118 with hypertension. After delivery to the operating room, all patients had sedation using either Dexmedetomidine or Versed with mild narcotic support. The standard thoracotomy technique was altered to perform procedures in a modified supine position. In case emergent intubation would be required, this position would facilitate control of airway. *The positioning of the patient was essentially supine with a small gel roll placed under the operative side to elevate the appropriate hemithorax. Incision and trocar placement varied depending on procedure performed/location of lesion. In general one single 10 mm. incision was used for the thoracoscope, and alongside the scope a grasper was placed through the same incision for manipulation of the lung. Secondary incisions were made based upon extent of procedure, but usual followed a standard VATS placement schematic.*

Only thirty patients had central venous catheters placed, and no swan-ganz catheters were used. Arterial catheters were used in 28 of the lobectomies and ten of the 68 decortications. In totally, 22 foley urinary catheters were used, all removed within six hours of completion of the procedure. Of the 132 patients who had a lung resection, 116 had extremely poor pulmonary function with forced expiratory volumes 1 second less than 0.8 (FEV_1_ < 0.8). One patient had FEV_1_ = 0.58. Thoracoscopic procedures were then performed. Any site that had a resection had application of Progel lung sealant applied. Prior to closure, a 28 French chest tube was placed. Post-operatively, the average chest tube duration was 1.2 days, ranging from 6 hours to 3.5 days.

There were no deaths in the series. Overall, only fourteen complications were seen in the series. There were ten atrial fibrillations from lobectomy patients and three from decortication patients. One patient had an intravenous site phlebitis. No patients had strokes or acute or delayed pneumothorax and there were no deep venous thrombosis, pulmonary emboli, urinary tract infections, pneumothoracies, pneumonia or readmission with 36 month follow-ups. We did not convert any patients to general anesthesia or intubate them during or after the procedures.

All patients were satisfactorily treated post-operatively with oral pain medication (Tylenol with Codeine) after one or two doses of intravenous morphine (2 mg every 2 hours). One hundred thirty-eight patients received intravenous or oral ketoralac. General patient satisfaction was high, with 98% of patients reporting a comfort index ≤ 1 on a scale of 0–10 with 0 being no pain and 10 being extreme pain. Two percent of patients reported a comfort index of 3–4.

## Discussion

The advantages of thoracic surgery performed under local anesthesia can be seen from our low post-operative complication rate and short mean hospital stays. Hazelriff et al. [7] looked at 1,820 VATS cases, almost all performed using a double lumen endotracheal tube under general anesthesia (around 1% of patients were treated under local anesthesia). Their wedge resections had an ALS of 5.1 days, as compared with 1.5 in our series. Their lobectomies and pleural biopsies had ALS of 6.3 and 5.8 days, respectively, while under local anesthesia, we experienced mean hospital stays of 1.8 and 1 days. Generally, we see that operating under local anesthesia more than halves the hospital stay of patients, thereby reducing costs and increasing patient satisfaction.

Junacovici et al. [8] studied 937 thoracic procedures, almost all of which were performed under general anesthesia. Local anesthesia was used in select patients with very poor cardiopulmonary status. The rate of postoperative complications in their study was 10.9%, with the most prevalent complication being air leaks. In comparison, our series under local anesthesia had a 4.8% incidence of postoperative complications. The use of Progel combined with meticulous surgical technique reduced our air leak duration to ≤24 hours with most stopping at 8 hours or less. In addition, 47 of their 937 patients required an excess period of drainage, up to 18 days, because of incomplete lung re-expansion. Using local anesthesia eliminates this issue because the lung is not collapsed in surgery and the patient is spontaneously breathing.

Junacovici et al. [8] found that older patients in poor condition or with malignancies were the highest contributors to the mortality rate. They suggested performing surgery under local anesthesia with little or no sedation in these patients to curb the mortality rate. As we put this suggestion into practice, we do find less complications and a lower mortality rate. More experience with VATS techniques and better instrumentation help to optimize results. As local anesthesia techniques are refined, we may also see lower complication and mortality rates as well.

## Conclusions

In our experience, the results of VATS under local anesthesia with sedation provide comparable, if not better, post-operative results to patients undergoing a general anesthetic. By choosing not to subject patients to general anesthesia with an endotracheal tube and one lung ventilation, we shortened the average hospital stay, provided quicker recovery times, better patient satisfaction and presumably obtained cost savings. Necessary equipment, personnel and patient positioning must be taken into consideration before beginning the procedure in the event that emergent intubation is required.

## Abbreviations

VATS: Video-assisted thoracic surgery
ALS: Average length of stay in hospital
